# Sustained virological response to peginterferon therapy in patients infected with HCV (genotypes 2 and 3), with or without HIV

**DOI:** 10.1186/1471-2334-14-S5-S4

**Published:** 2014-09-05

**Authors:** Silvia Odolini, Silvia Amadasi, Carlo Cerini, Mariarosaria Giralda, Paola Nasta, Francesco Castelli

**Affiliations:** 1 University Division of Infectious and Tropical Diseases, University of Brescia and Spedali Civili General Hospital, Brescia, Italy

**Keywords:** HIV, HCV, coinfection, SVR, anti-HCV treatment, peginterferon, ribavirin

## Abstract

**Background:**

HIV infection leads to a faster progression of liver disease in subjects infected with HCV, as compared with HCV mono-infected patients. Previous reports suggest that sustained virological response (SVR) rates are lower in HIV/HCV coinfection than in HCV monoinfection. We aimed to compare SVR rates of these two populations.

**Methods:**

We retrospectively analyzed clinical, biochemical and virological data of HCV and HIV/HCV infected patients with HCV genotypes 2 and 3 who started anti-HCV treatment between March 2004 and November 2012, at a single large center. Intention-to-treat (ITT) and per-protocol (PP) analysis were performed. Univariate and multivariate logistic regression analyses were performed to assess predictors of SVR.

**Results:**

461 patients were analyzed: 307 (66.6%) males, 76 (16.5%) infected with HIV. Several differences at baseline between HCV monoinfected and HIV/HCV coinfected patients were observed. HCV monoinfected group was characterized by higher prevalence of genotype 2 (53% *vs *5.3%), higher baseline HCV viral load (50% *vs *35%), shorter mean duration of treatment (19 *vs *41 weeks), more frequent use of peginterferon alfa-2a (84.5% *vs *69.7%), lower prevalence of cirrhosis (6% *vs *31.6%). Globally, SVR was achieved by 353 (76.6%) patients and 321 (83.8%) in the PP analysis. No statistically relevant differences were found in SVR rates between the two groups, either in ITT [78.2% (n = 301/385) *vs *68.4% (n = 52/76), *p =*0.066, respectively] than in PP analysis [83.6% (n = 276/330) *vs *84.9% (n = 45/53), *p = *0.8].

**ITT analysis**

At univariate and multivariate analysis, baseline HCV-RNA >500.000 IU/ml [OR 0.4 (0.24-0.66), *p *= 0.0004], use of peginterferon alfa-2b [OR 0.5 (0.27-0.93) *p *= 0.033], platelets count <130.000/mm^3 ^[OR 0.45 (0.2-0.99), *p *= 0.045], interruption of peginterferon therapy [OR 0.2 (0.1-0.4), *p*<0.0001], interruption of ribavirin treatment [OR 0.34 (0.17-0.69), *p *= 0.0026] were related with lower rate of SVR.

**PP analysis**

Only HCV-RNA >500.000 IU/ml and interruption of ribavirin were related to lower probability to achieve SVR at both univariate and multivariate analysis [OR 0.41 (0.23-0.75), *p *= 0.004; OR 0.24 (0.1-0.5), *p *= 0.0004, respectively].

**Conclusions:**

Higher baseline viral loads and interruption of peginterferon and/or ribavirin were associated with a poor outcome of anti-HCV treatment while HIV infection was not related to major or minor probability to achieve SVR.

## Background

Hepatitis C virus (HCV) infection is highly prevalent (20-40%) in subjects infected with the human immunodeficiency virus (HIV) because of common transmission pathways [1]. HIV/HCV coinfected subjects have higher HCV viral load, faster progression to cirrhosis, higher incidence of liver decompensation and liver related deaths when compared to HCV monoinfected patients [2,3]. The primary goal of HCV therapy is to cure the infection, which results in the elimination of the virus after cessation of treatment in a variable proportion of patients according to genotype [4]. It has been observed that eradication of HCV in HIV/HCV coinfected patients is associated not only with a reduction in liver-related complications and mortality, but also with a reduction in HIV progression and mortality, even not related to liver disease [5]. The standard treatment for chronic HCV infection is the combination of peginterferon alfa (PegIFN) plus ribavirin (RBV) for at least 24 weeks [6]. A shortened duration of treatment (12-16 weeks) can be considered in patients with HCV genotypes 2 or 3, low baseline HCV-RNA levels and mild fibrosis, who achieved undetectable HCV-RNA at week 4 of treatment (rapid virological response, or RVR). Sustained virological response (SVR) is rather defined as an undetectable HCV-RNA level (<50 IU/ml) 24 weeks after treatment withdrawal, and it indicates the good outcome of treatment. In HIV/HCV coinfection the minimal duration of anti-HCV treatment is 48 weeks. Monoinfected patients with positive predictors of response, as listed above, may only need 24 weeks of therapy [4]. The likelihood of achieving SVR is lower in HIV/HCV coinfected persons than in those with HCV monoinfection [7-9]. The rate of SVR in HCV monoinfected patients varies between 46-55% for genotypes 1 or 4 and 76-88% for genotypes 2 or 3 [10,11] while in HIV/HCV coinfected subjects it varies between 14-38% for genotypes 1 or 4 and 43-73% for genotypes 2 or 3 [6-9].

Moreover, for HCV monoinfection, it has been described that the percentage of SVR is higher in patients treated with PegIFN alfa-2a plus ribavirin than in those who received PegIFN alfa-2b plus ribavirin, particularly for genotypes 1 and 4 [10,12].

In this study we aimed to compare the prevalence of SVR in HCV monoinfected and HIV/HCV coinfected patients with HCV genotypes 2 and 3 and to determine SVR related variables according to Intention to Treat (ITT) and Per Protocol (PP) analysis.

## Methods

### Patients and data collection

A retrospective, single-center, observational study was conducted in the University Division of Infectious and Tropical Diseases, Spedali Civili General Hospital in Brescia, Italy. The enrolled sample was represented by HCV-infected patients who started anti-HCV treatment between March 2004 and November 2012. Diagnosis of chronic HCV infection was defined as positive anti-HCV antibodies and detectable HCV-RNA at baseline. All patients were ≥18 years old and naïve to previous anti-HCV treatment. Only patients with genotypes 2 or 3 were considered for this study, since these are still candidates only for dual treatment. On the other hand, in our experience there were a few HIV patients with HCV genotypes 1 and 4 eligible for treatment, so the two groups in the study (HCV monoinfected and HIV/HCV coinfected) would be not comparable.

Patients that presented with any of the following criteria at the baseline evaluation were excluded: HBsAg positivity; pregnancy or lactation; hypersensitivity to the prescribed drugs or to their excipients; autoimmune hepatitis; severe liver disease (Child-Pugh score ≥6) or previous diagnosis of hepatocellular carcinoma; history of severe cardiovascular disease, including unstable cardiac disease in the 6 months preceding the baseline visit; severe psychiatric disease or inability to follow a plan of appointed medical examinations; history of actual alcohol abuse; previous exposure to anti-HCV drugs. Advanced liver disease at the ultrasound scan identified cirrhotic patients. At the beginning of therapy, PegIFN alfa-2a (Pegasys^®^, Roche) or PegIFN alfa-2b (PegIntron^®^, Schering-Plough) and RBV capsules (Rebetol^®^, Schering-Plough) or tablets (Copegus^®^, Roche) were prescribed. The choice of the drug and of the dosage depended on the investigators who based their decision on product and patient characteristics and on available scientific evidence at the time of enrolment and during follow-up.

At baseline and during the period of treatment, demographic, clinical and laboratory data were collected and recorded in an internal electronic chart (Netcare health system^®^). The study was conducted under the provisions of the Declaration of Helsinki, and in accordance with the International Conference on Harmonization Consolidated Guideline on Good Clinical Practice.

Since as this study was retrospective and non-pharmacological, written informed consent has not been provided. In Italy, ethical authorization for these studies is not necessary (see Italian Guidelines for classification and conduction of observational studies, established by the Italian Drug Agency, "Agenzia Italiana del Farmaco - AIFA" on March 20^th^, 2008). Moreover, for this study we used the general authorization of the Italian Guarantor for the use of demographical and clinical data of patients who signed Spedali Civili General Hospital of Brescia informed consent, at the time of the admission in our outpatient Clinic.

### Statistical analysis

All patients with genotype 2 or 3 recorded in the database and exposed to PegIFN plus RBV were retrospectively evaluated in order to assess factors associated with SVR. Viral load was also registered at week 4, in order to identify RVR, and at week 12 of treatment in all patients.

An ITT analysis including all patients who received at least one dose of anti-HCV treatment was performed. All patients who discontinued therapy because of adverse events or voluntary withdrawal or virological failure or any other cause were included in this analysis. Only subjects who were lost to follow-up, as patients with a missing HCV-RNA concentration for any reason at the end of follow-up, were excluded from the analysis. With the same inclusion criteria, a PP analysis, including only patients who concluded the standardised anti-HCV cycle of therapy, according to current Guidelines, was also planned.

The main characteristics of the study population were described using proportions or central tendency and variability indicators in case of qualitative and quantitative variables, respectively. Comparisons between groups for categorical variables were carried out using Chi-square or Fishers's exact tests, as appropriate, while Wilcoxon-Mann-Whitney U-test was used with mean values for individual variables. Univariate and multivariate logistic regression analyses were performed to assess the predictors of SVR, including potentially confounding variables such as: age, gender, genotype and severity of hepatic impairment. All *p *values were two-tailed, and were considered significant only when below 0.05. All analyses were performed using Epi Info software toll (version 3.5.3.; Center of Disease Control, Atlanta, USA).

## Results

### Study population and descriptive analysis

During the study period, 461 patients with HCV genotype 2 or 3 infection were registered: 307 (66.6%) males and 154 (33.4%) females. There were 76 (16.5%) patients infected with HIV. The baseline characteristics of the two groups are shown in Table 1. 75/76 patients with HIV (98.7%) were in treatment with highly-active antiretroviral therapy (HAART) at baseline, and 69 of them (90.8%) showed undetectable HIV-RNA values. The mean value of CD4+ T lymphocytes was 489 cell/mm3 at baseline, while the mean nadir value was 213 cell/mm3.

**Table 1:** Descriptive analysis of baseline clinical and therapeutical variables: monoinfected vs coinfected patients.

Variables	HIV- (n = 385)	HIV+ (n = 76)	OR (95% IC)	*p*-value
Cirrhosis, % (n)	6% (23/385)	31.6 % (24/76)	7.2 (3.7-13.8)	<0.0001

HCV-RNA >500.000 IU/ml, % (n)	50.1% (191/381)	35.5 % (27/76)	0.5 (0.3-0.9)	0.02

PegIFN alfa-2b vs alfa-2a, % (n)	15.5% (61/385)	30.3 % (23/76)	2.34 (1.3-4.1)	0.002

PegIFN reduction, % (n)	11.7% (45/385)	5.3 % (4/76)	0.4 (0.1-1.1)	0.09

Stop PegIFN, % (n)	14% (54/385)	30.3 % (23/76)	2.6 (1.5-4.6)	0.0005

RBV reduction, % (n)	14.5% (56/385)	14.5 % (11/76)	0.9 (0.4-1.9)	0.987

Stop RBV, % (n)	9.6% (37/385)	27.6 % (21/76)	3.5 (1.9-6.5)	<0.0001

Age >45 years, % (n)	51.9% (200/385)	50% (38/76)	0.92 (0.5-1.5)	0.76

Male gender, % (n)	63.4% (244/385)	82.9 % (63/76)	2.8 (1.5-5.4)	0.009

Genotype 3, % (n)	47% (181/385)	94.7 % (72/76)	20.2 (7.8-66.2)	<0.0001

PLT <130,000 cell/mm^3^, % (n)	7.5% (29/385)	32.9 % (25/76)	6 (3.2-11)	<0.0001

HIV RNA <50 cp/ml, % (n)		90.8% (69/76)		

Patients in HAART, % (n)		98.7% (75/76)		

**Variables**	**HIV- (n = 385)**	**HIV+ (n = 76)**		***p*-value**

ALT, IU/l, mean (DS)	117.6 (111.4)	114.6 (74.7)		0.2126

AST, IU/l, mean (DS)	69.2 (66.3)	74.8 (49.1)		0.0123

BMI, mean (DS)	25.2 (4.2)	23.7 (3.1)		0.006

Cholesterol, mg/dl, mean (DS)	175 (42.7)	150.5 (40)		<0.0001

Triglycerids, mg/dl, mean (DS)	88.8 (43.8)	112.3 (63)		0.001

Age, years, mean (DS)	46.1 (12.3)	44 (5.5)		0.33

Glycemia, mean (DS)	94.4 (19.1)	90.8 (12.2)		0.082

Hb, g/dl, mean (DS)	14.9 (1.5)	14.5 (2.1)		0.095

PT, mean (DS)	103.6 (11.4)	100.5 (13.4)		0.12

Neutrophils, cell/mm^3^, mean (DS)	3400 (1231)	3080 (1342)		0.014

Weeks of treatment, mean (DS)	19 (8)	41 (17)		<0.0001

Bilirubin, mg/dl, mean (DS)	0.7 (0.4)	1.5 (1.4)		<0.0001

Albumin, g/dl, mean (DS)	4.5 (2.2)	4.4 (0.4)		0.896

CD4+ cell count (cell/mm3), mean (DS)		489 (273)		

Nadir CD4 (cell/mm3), mean (DS)		213 (158)		

PLT, platelets; ALT, alanine aminotransferase; AST, aspartate aminotransferase; BMI, Body Mass Index; Hb, hemoglobin; PT, prothrombin time.

Monoinfected and coinfected populations at baseline differed from each other in distribution of genotype 3 (respectively, 47% and 94.7%, *p*<0.0001), male gender (63.4% vs 82.9%, *p *= 0.009) and prevalence of cirrhosis (6% *vs *31.6%, *p*<0.0001). Baseline bilirubin (0.7 *vs *1.5 mg/dl, *p*<0.0001), cholesterol (175 *vs *150 mg/dl, *p *= 0.006), triglycerides (88.8 *vs *112.3 mg/dl, *p *= 0.001), neutrophils (3400 *vs *3080 cell/mm^3^, *p*<0.014) and BMI (25.2 *vs *23.7, *p *= 0.006) mean values were also different between the two groups (Table 1). Mono-infected patients were more likely to use PegIFN alfa-2a (84.5% *vs *69.7%, *p *= 0.002). A lower percentage of patients with HIV infections had HCV-RNA viral load higher than 500.000 IU/ml at the beginning of the anti-HCV treatment, if compared to patients without HIV infection [35% *vs *50%; OR = 0.5 (0.3-0.9), *p *= 0.02]. Moreover, during the follow up period, HIV patients showed longer duration of treatment (41 *vs *19 weeks, *p*<0.0001) as well as higher probability to stop specific anti-HCV drugs for any reason (30% *vs *14%, *p *= 0.0005, for PegIFN and 27.6% *vs *9.6%, *p*<0.0001, for RBV).

### Prevalence of SVR

**Figure 1:**
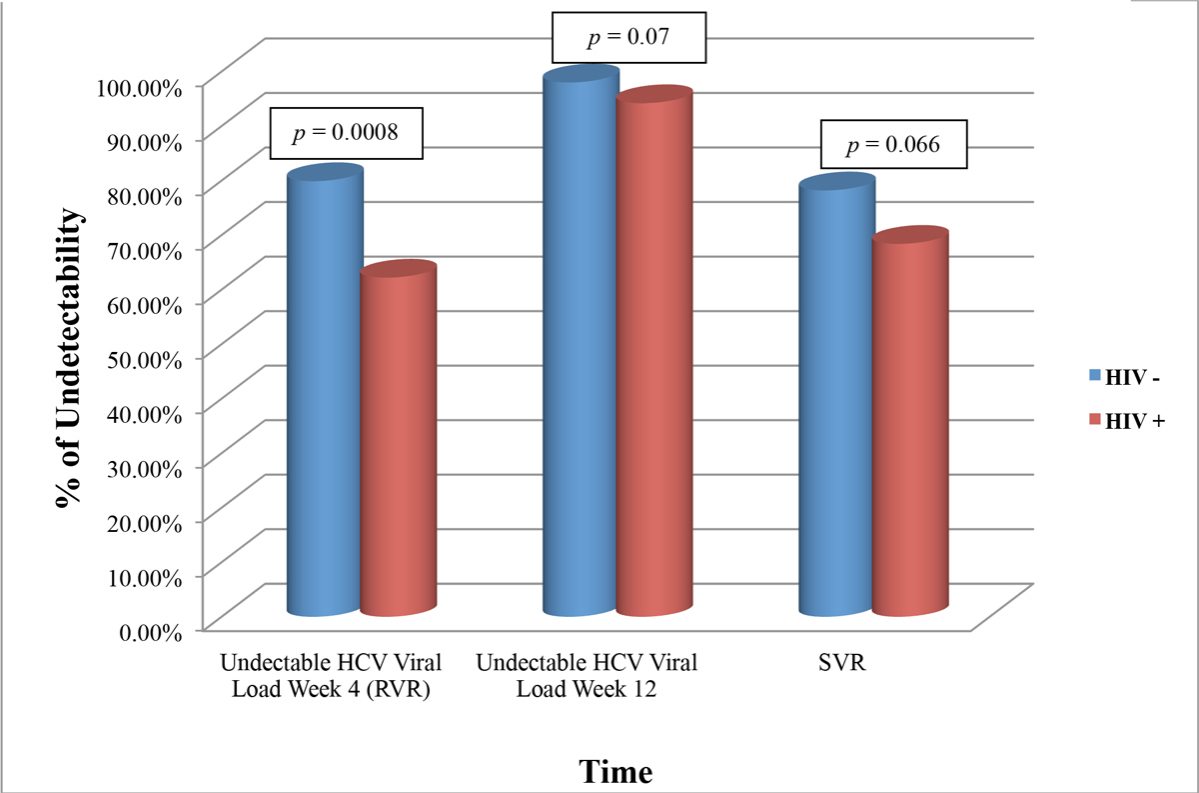
**Rate of undetectability in monoinfected and coinfected patients (ITT analysis)**.

**Figure 2:**
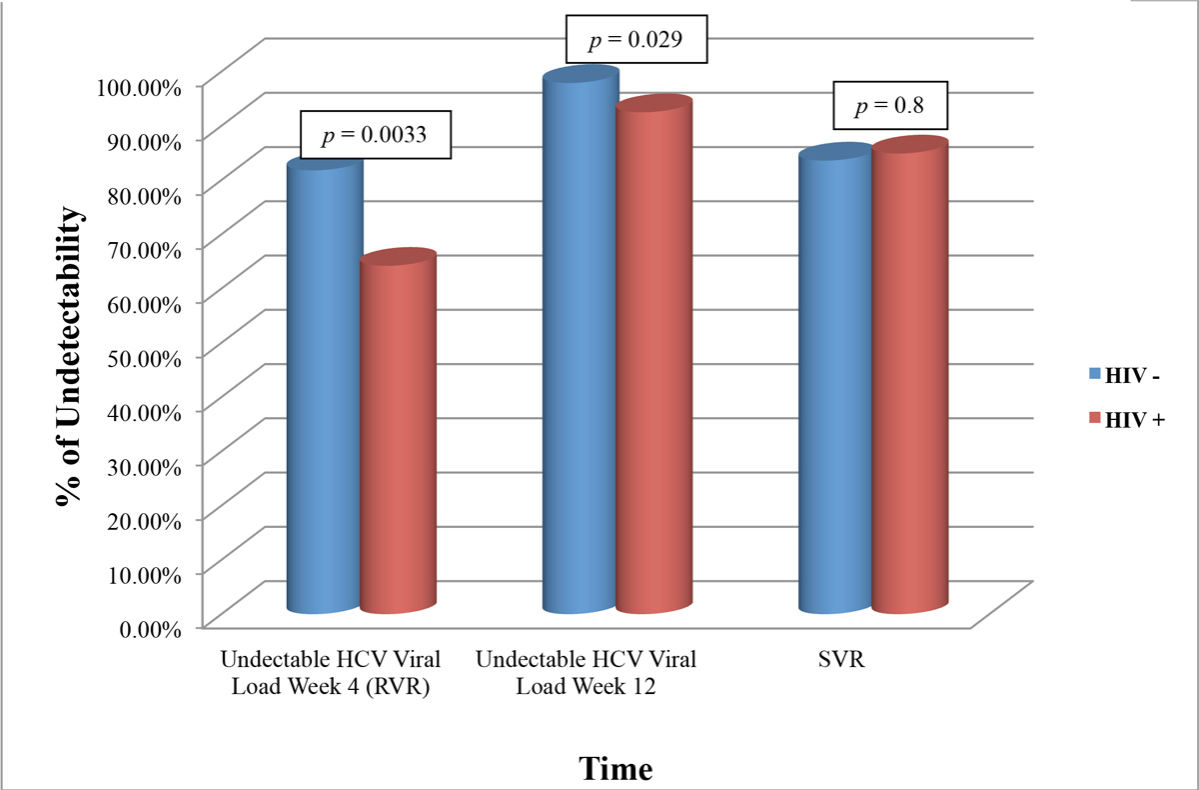
**Rate of undetectability in monoinfected and coinfected patients (PP analysis)**.

In the ITT analysis, SVR was achieved by 353 patients (76.7%), while in the PP analysis SVR was achieved by 321 patients (83.8%), globally. As reported in figure 1 and 2, no statistically relevant differences were found in SVR rate between mono and coinfected patients, either in ITT [78.2% (n = 301/385) *vs *68.4% (n = 52/76)] and in PP analysis [83.6% (n = 276/330) *vs *84.9% (n = 45/53)]. As expected, subjects without HIV infection showed a higher percentage of RVR achieved either in the ITT (79.9%, n = 299/374, *p *= 0.0009) and in the PP analysis (81.7%, n = 269/329; *p *= 0.0033). After 12 weeks of treatment, the rate of undetectability was similar in monoinfected and coinfected patients in ITT analysis, but it was statistically different in the PP analysis (97.9% *vs *92.5%, *p *= 0.029).

### Predictors of SVR in ITT analysis

At univariate analysis, baseline HCV-RNA >500.000 IU/ml [OR 0.5 (0.3-0.7) *p *= 0.0009], use of PegIFN alfa-2b instead of PegIFN alfa-2a [OR 0.5 (0.3-0.9), *p *= 0.014], platelets count <130.000/mm^3 ^[0.3 (0.2-0.6), *p *= 0.0004], interruption of PegIFN therapy [OR 0.1 (0.1-0.2), *p*<0.0001], interruption of RBV treatment [OR 0.3 (0.2-0.5), *p*<0.0001], lower duration of treatment [20 *vs *24 weeks, *p *= 0.0006] are related with a lower rate of SVR (Table 2).

**Table 2:** Variables related with SVR at univariate and multivariate analysis (ITT).

	Univariate Analysis			Multivariate Analysis	
**Variables**	**OR** **(IC 95%)**		***p *value**	**OR** **(IC 95%)**	***p *value**

HCV-RNA >500.000 IU/ml	0.5 (0.3-0.7)		0.0009	0.39 (0.24-0.66)	0.0004

PegIFN alfa-2b vs alfa-2a	0.5 (0.3-0.9)		0.014	0.5 (0.27-0.93)	0.033

Stop PegIFN	0.1 (0.1-0-2)		<0.0001	0.19 (0.1-0.4)	<0.0001

Stop RBV	0.3 (0.2-0.5)		<0.0001	0.34 (0.17-0.69)	0.0026

PLT <130.000 cell/mm^3^	0.35 (0.2-0.6)		0.0004	0.45 (0.2-0.99)	0.048

Cirrhosis	0.6 (0.3-1.2)		0.15	1.37 (0.57-3.3)	0.47

Age >45 years	0.7 (0.45-1)		0.11	0.71 (0.4-1.26)	0.25

HIV infection	0.6 (0.4-1.1)		0.066	0.85 (0.32-2.23)	0.74

RBV reduction	1.5 (0.8-2.9)		0.25		

PegIFN reduction	1.2 (0.6-2.6)		0.59		

Gender (male vs female)	0.6 (0.4-0.9)		0.34	0.64 (0.36-1.14)	0.13

HCV genotype 2 vs 3	0.75 (0.48-1.16)		0.2	0.97 (0.5-1.8)	0.93

**Variables**	**SVR no**	**SVR yes**	***p *value**	**OR** **(IC 95%)**	***p *value**

Weeks of treatment, mean (SD)	20.3 (15.4)	24.3 (12.2)	0.0006	1 (0.9-1.1)	0.22

The multivariate analysis confirmed HCV-RNA >500.000 IU/ml [OR 0.4 (0.24-0.66), *p *= 0.0004], interruption of PegIFN therapy [OR 0.2 (0.1-0.4), *p*<0.0001], interruption of RBV treatment [OR 0.34 (0.17-0.69), *p *= 0.0024], thrombocytopenia [OR 0.45 (0.2-0.99), *p *= 0.045] and use of PegIFN alfa-2b [OR 0.05 (0.27-0.93), *p *= 0.033], as factors significantly related to a poor outcome to anti-HCV treatment.

### Predictors of SVR in PP analysis

Performing the PP analysis (Table 3), only HCV-RNA >500.000 IU/ml [OR 0.4 (0.2-0.7), *p *= 0.0019] and interruption of RBV [OR 0.25 (0.1-0.5), *p *= 0.0001] are related to lower probability to achieve SVR at univariate analysis. This data is confirmed at the multivariate analysis.

**Table 3:** Variables related with SVR at univariate and multivariate analysis (PP).

	Univariate analysis			Multivariate analysis	
**Variables**	**OR** **(IC 95%)**		***p *value**	**OR** **(IC 95%)**	***p *value**

HCV-RNA >500.000 IU/ml	0.4 (0.2-0.7)		0.0019	0.41 (0.23 - 0.75)	0.004

PegIFN alfa-2b vs alfa-2a	0.58 (0.3-1.15)		0.098	0.48 (0.24-0.97)	0.041

Stop RBV	0.25 (0.1-0.5)		0.0001	0.24 (0.1-0.5)	0.0004

PLT <130.000 cell/mm^3^	0.5 (0.2-1.3)		0.1575	0.83 (0.3-2.2)	0.715

Cirrhosis	0.6 (0.3-1.7)		0.3616		

Age >45 years	0.7 (0.4-1.2)		0.1868	0.73 (0.4-1.34)	0.31

HIV infection	1.1 (0.5-2.6)		0.8159	1.87 (0.49-7.12)	0.36

RBV reduction	1.7 (0.7-4.6)		0.2287		

PegIFN reduction	0.9 (0.4-2.2)		0.8685		

Gender (male vs female)	0.7 (0.3-1.2)		0.1868	0.67 (0.3-1.3)	0.24

HCV genotype 2 vs 3	1 (0.6-1.8)		0.9		

**Variables**	**SVR no**	**SVR** **yes**	**p value**	**OR** **(IC 95%)**	***p *value**

Weeks of treatment, mean (SD)	27 (14)	25 (12)	0.18	0.98 (0.947-1)	0.39

HIV infection does not seem to be related to major or minor probability to achieve SVR, in particular if patients are controlled for age, gender, HCV genotype, baseline HCV viral load, presence of cirrhosis and length of treatment.

### HCV genotype 3 analysis

A further analysis was performed excluding all patients with HCV genotype 2, given the difference of genotype distribution between mono and coinfected populations (53% *vs *5.3%, see Table 1). As reported in Table 4 this analysis, including 253 patients, confirms that baseline HCV viral load >500.000 IU/ml is more frequent in HIV negative than in HIV positive patients [46.9% *vs *31.9%, OR 0.53 (IC 95% 0.3-0.9), *p =*0.03]. Moreover, the probability to achieve SVR in monoinfected and coinfected patients is not different either in ITT than in PP analysis (Table 4). Multivariate analyses, performed both in ITT and PP scheme, confirm that HIV infection is not a factor influencing the success of anti-HCV treatment (data not shown), if patients are equalized for age, gender, type of PegIFN, hepatic impairment and length of treatment.

**Table 4:** Descriptive analysis of baseline clinical and therapeutical variables : monoinfected vs coinfected patients with HCV genotype 3.

Variables	HIV- (n = 181)	HIV+ (n = 72)	Odds Ratio (95% IC)	*p*-value
Cirrhosis, % (n)	66% (12)	30.6% (22)	6.1 (2.8-13.6)	<0.0001

HCV-RNA >500.000 IU/ml, % (n)	46.9% (84)	31.9% (23)	0.53 (0.3-0.9)	0.03

PegIFN alfa-2b, % (n)	23.2% (42)	29.2% (21)	1.36 (0.7-2.5)	0.32

PegIFN reduction, % (n)	11.6% (21)	5.6% (4)	0.45 (0.13-1.3)	0.15

Stop PegIFN % (n)	16.6% (30)	31.9% (23)	2.35 (1.24-4.44)	0.007

RBV reduction, % (n)	12.2% (22)	13.9% (10)	1.6 (0.5-2.6)	0.71

Stop RBV, % (n)	9.4% (17)	29.2% (21)	3.95 (1.93-8.17)	<0.0001

Male gender, % (n)	76.8% (139)	81.9% (59)	1.37 (0.7-2.8)	0.37

Age >45 years, % (n)	27.6% (50)	48.6% (35)	2.4 (1.4-4.4)	0.001

PLT <130,000 cell/mm^3^, % (n)	8.3% (15)	33.3% (24)	5.4 (2.7-11.5)	<0.0001

**Variables**	**HIV- (n = 181)**	**HIV+ (n = 72)**		***p*-value**

ALT, mean (DS)	139.7 (118.6)	117.6 (75.5)		0.26

AST, mean (DS)	78.7 (70.6)	76.5 (49.9)		0.69

BMI, mean (DS)	25.1 (3.7)	23.6 (3.2)		0.006

Cholesterol, mean (DS)	152.5 (39.3)	148.9 (40)		0.52

Triglycerids, mean (DS)	82.3 (45)	108.5 (61.8)		0.0004

Glycemia, mean (DS)	93.6 (21.9)	90.2 (12.1)		0.39

Hb, mean (DS)	15.3 (1.5)	14.5 (2.1)		0.003

PT, mean (DS)	102 (11.6)	101 (12.6)		0.96

Neutrophils, mean (DS)	3597 (1327)	3118 (1364)		0.004

Weeks of treatment, mean (DS)	20.7 (9.1)	40.7 (17.8)		<0.0001

Bilirubine, mean (DS)	0.82 (0.45)	1.55 (1.48)		0.0002

Albumine, mean (DS)	4.63 (3.13)	4.39 (0.47)		0.47

**SVR rate, ITT analysis**	**HIV- (n = 181)**	**HIV+ (n = 72)**	**Odds Ratio** **(95% IC)**	***p*-value**

SVR, % (n)	76.8 % (139)	68.1 % (72)	0.65 (0.3-1.2)	1.15

**SVR rate, PP analysis**	**HIV- (n = 151)**	**HIV+ (n = 49)**	**Odds Ratio** **(95% IC)**	***p*-value**

SVR, % (n)	83.4 % (126)	85.7 % (42)	1.19 (0.5-3.1)	0.7

PLT, platelets; ALT, alanine aminotransferase; AST, aspartate aminotransferase; BMI, Body Mass Index; Hb, hemoglobin; PT, prothrombin time.

## Discussion

In our study similar rates of SVR were observed both in HCV monoinfected and HIV/HCV coinfected patients suggesting, as reported in the multivariate analysis, that HIV infection does not affect the probability to achieve the SVR, even if HIV patients needed to be treated for longer.

It is known that HIV/HCV coinfected patients are characterized by a reduced likelihood of HCV clearance and higher levels of baseline HCV-RNA [13]. Despite this, in our study population, HCV monoinfected patients were more likely to have higher baseline HCV viral load. HAART seems not to affect HCV-RNA levels, even if its potential effect on the natural history of chronic HCV disease is not clear [6].

Although a large scale US trial showed no significant differences of efficacy between PegIFN alfa-2a and alfa-2b [14], our data revealed higher rates of SVR in patients treated with PegIFN alfa-2a. Several studies confirmed our finding, particularly for genotype 1 and 4 [10,12,15]. However there is currently no conclusive evidence that one PegIFN should be preferred as first-line therapy [4].

According to EASL guidelines [4], the dose and duration of therapy is considered one of the strongest predictors of SVR. In our study, treatment drop out was observed in 14% of monoinfected patients and 30.3% of coinfected patients and, respectively the 9.6% and the 27.6%, experienced RBV drop out. Interruption of RBV treatment resulted related to poor antiviral treatment outcome both in ITT and PP analysis. This finding has already been reported [16,17]. The RBV starting dose was the same for all groups, according to current guidelines (15 mg/kg/die) and there were no difference in RBV reduction dose between mono and coinfected patients. However, SVR was reduced in patients who discontinued combination therapy as well as in those taking reduced RBV dose (<60%) in a retrospective analysis on HCV genotype 1b patients [16]. In an open randomized multicentre Italian trial that aimed to compare the efficacy and tolerability of PegIFN plus RBV or PegIFN monotherapy in HIV/HCV positive patients undergoing HAART, higher rates of SVR were achieved with the combination therapy confirming this regimen as a solid option for the treatment of HIV/HCV coinfected patients [17]. This highlights the importance of a full course of combination treatment even in patients already taking high daily intake of pills, as HIV infected patients.

Compensated liver cirrhosis is known to be a predictor of worse response to PegIFN plus RBV in HCV-monoinfected patients [18,19]. According to this, a prospective cohort study on 841 HIV/HCV coinfected patients reported lower efficacy of PegIFN plus RBV in cirrhotic patients compared to those without cirrhosis, although a substantial rate of SVR was achieved in HCV genotype 3 patients even in the presence of cirrhosis [20]. In our study liver biopsy was not available for all patients within the year before starting treatment and therefore, diagnosis of cirrhosis was based on ultrasound features. Despite this, thrombocytopenia, a common marker of advanced liver disease, was associated with lower rates of SVR highlighting the importance of early treatment of HCV infection. This has been observed also in a retrospective study where significant lower rates of SVR were achieved in treatment-naive patients, if treated for 48 weeks, with baseline platelet counts below 130000/μL [21].

Some limitations need to be considered when interpreting this result. Primarily, baseline characteristics of the two groups are different with a smaller number of HIV infected patients and secondly, diagnosis of cirrhosis was based only on ultrasound parameters and not on histological findings. Furthermore, duration of treatment is different between the two groups and the use of supportive therapies, as well as the use of granulocyte colony-stimulating factor (G-CSF), is to date permitted in Italy only in HIV infected patients, promoting the maintenance of full treatment regimens despite the occurrence of neutropenia.

Nevertheless, our results strongly support an early anti-HCV treatment also in HIV/HCV coinfected patients. Up to now, only a small part of coinfected patients receive an anti-HCV treatment. This is because of patients' fragility due to a perceived inclination to poor compliance and intolerance to the treatment, metabolic alterations, hematological toxicity and HAART interactions. All these factors have led to a low eligibility to anti-HCV treatment for this group of patients, but a right counseling and setting should be enhanced in order to reduce the number of early discontinuation of treatment, particularly if adverse events occur.

## Conclusions

As HIV infection seems not related to major or minor probability to achieve SVR, an early treatment of HIV/HCV coinfected patients should always be considered in order to avoid the progression of liver disease. A strict adherence and follow-up and a proper management of side effects are essential elements for SVR achievement.

## List of abbreviations

HCV, hepatitis C virus; HIV, human immunodeficiency virus; PegIFN, peginterferon; RBV, ribavirin; RVR, rapid virological response; SVR, sustained virological response; ITT, Intention to Treat; PP, Per Protocol; HAART, highly-active antiretroviral therapy; G-CSF, granulocyte colony-stimulating factor; PLT, platelets; ALT, alanine aminotransferase; AST, aspartate aminotransferase; BMI, Body Mass Index; PLT, platelets; Hb, hemoglobin; PT, prothrombin time.

## Competing interests

PN received grants for speeches and attendance to Conferences from Bristol-Myers Squibb, Janssen, Viiv Healthcare, Merck-Sharp & Dohme. FC acts as principal investigator in clinical trials sponsored by BMS, Janssen, Roche, Merck-Sharp & Dohme and Novartis and he received research grant from Pfizer, Viiv and Abbott. CC received grants from Gilead Sciences for research projects. All the other authors declare that they have no competing interests.

## Authors' contributions

Study concept and design: PN, SO, SA, CC, MG. Acquisition of data: CC, MG. Analysis and interpretation of data: CC, MG. Drafting of the manuscript: SO, SA, CC, MG. Critical revision of the manuscript for important intellectual content: PN, FC. All authors read and approved the final manuscript.

## Declarations

Publication of this supplement was partly supported by an unrestricted grant provided by Roche. The articles were independently prepared by the authors with no input from Roche. Roche were not involved in selecting the articles for the supplement. The pegylated IFN/pegIFN treatment mentioned in this article is produced by Roche.

This article has been published as part of *BMC Infectious Diseases *Volume 14 Supplement 5, 2014: Proceedings of the Third Workshop of the HCV Study Group in the Calabria Region: Results from the South Italian Network for Rational Guidelines and International Epidemiology (SINERGIE) Project. The full contents of the supplement are available online at http://www.biomedcentral.com/bmcinfectdis/supplements/14/S5.
